# The energy disruptor metformin targets mitochondrial integrity via modification of calcium flux in cancer cells

**DOI:** 10.1038/s41598-017-05052-2

**Published:** 2017-07-11

**Authors:** Camille Loubiere, Stephan Clavel, Jerome Gilleron, Rania Harisseh, Jeremy Fauconnier, Issam Ben-Sahra, Lisa Kaminski, Kathiane Laurent, Stephanie Herkenne, Sandra Lacas-Gervais, Damien Ambrosetti, Damien Alcor, Stephane Rocchi, Mireille Cormont, Jean-François Michiels, Bernard Mari, Nathalie M. Mazure, Luca Scorrano, Alain Lacampagne, Abdallah Gharib, Jean-François Tanti, Frederic Bost

**Affiliations:** 1 Inserm U1065, C3M, Team Cellular and Molecular Physiopathology of Obesity and Diabetes, Nice, France; 2 Université Nice Côte d’Azur, Inserm, Nice, France; 3 Inserm U1060/ INRA 1235/ Université-Lyon1/ INSA, Lyon, France; 4 Inserm U1046, UMR CNRS 9214, Université de Montpellier, Montpellier, France; 5 Northwestern University, Chicago, IL USA; 6 Department of Biology, University of Padua, Padua, Italy; 7 Dulbecco-Telethon Institute, Venetian Institute of Molecular Medicine, Padua, Italy; 8 Centre Commun de Microscopie Appliquée, Université de Nice Sophia-Antipolis, Nice, France; 9 Centre Hospitalier Universitaire (CHU) de Nice, Hôpital Pasteur, Laboratoire Central d’Anatomo Pathologie, 06002 Nice, France; 10 Inserm U1065, C3M, Team Biology and pathology of melanocyte cells: From skin pigmentation to melanomas, Nice, France; 11 CNRS, Institute of Molecular and Cellular Pharmacology, Sophia Antipolis, France; 12 Institute for Research on Cancer and Aging of Nice, CNRS-UMR 7284-Inserm U1081, University of Nice Sophia-Antipolis, Centre Antoine Lacassagne, Nice, France

## Abstract

Mitochondrial integrity is critical for the regulation of cellular energy and apoptosis. Metformin is an energy disruptor targeting complex I of the respiratory chain. We demonstrate that metformin induces endoplasmic reticulum (ER) stress, calcium release from the ER and subsequent uptake of calcium into the mitochondria, thus leading to mitochondrial swelling. Metformin triggers the disorganization of the cristae and inner mitochondrial membrane in several cancer cells and tumors. Mechanistically, these alterations were found to be due to calcium entry into the mitochondria, because the swelling induced by metformin was reversed by the inhibition of mitochondrial calcium uniporter (MCU). We also demonstrated that metformin inhibits the opening of mPTP and induces mitochondrial biogenesis. Altogether, the inhibition of mPTP and the increase in mitochondrial biogenesis may account for the poor pro-apoptotic effect of metformin in cancer cells.

## Introduction

Maintaining mitochondrial structural integrity is essential for cells to produce energy, overcome environmental stresses such as nutrient deprivation and hypoxia and respond to genotoxic agents, including chemotherapy. Consequently, the disruption of mitochondrial metabolism sensitizes cells to apoptosis and opens new therapeutic avenues in cancer treatment. Metformin is a biguanide and a widely prescribed anti-diabetic agent, but it is also a metabolic disruptor that specifically targets the metabolism of cancer cells^1^. Several reports have shown that this drug inhibits cancer cell growth and has antitumoral effects^2, 3^. Biguanides inhibit the activity of the mitochondrial respiratory chain complex I, thus leading to energetic stress due to the decrease in ATP synthesis^4–6^. Decreased intracellular ATP leads to the activation of the energy sensitive kinase AMP-activated protein kinase (AMPK) and the inhibition of mechanistic target of rapamycin (mTORC1), which regulates cell growth and cell proliferation and is frequently hyperactivated in tumors. Despite the well-characterized effects of biguanides on the respiratory chain, their effects on the mitochondrial ultrastructure are poorly understood.

Calcium is a central regulator of mitochondrial metabolism; it regulates the activity of several enzymes in the tricarboxylic acid cycle and participates in the control of energy metabolism of cells^7^. Calcium concentration is tightly regulated to allow for precise biological processes such as muscle contraction, apoptosis or cell proliferation. The endoplasmic reticulum (ER) is the main intracellular organelle that stores calcium. IP3 (inositol 1,4,5-trisphosphate) binds to its receptor IP3 receptor (IP3R) and is responsible for the release of calcium from the ER. The mitochondria act as cytosolic calcium buffers, and calcium uptake into mitochondrion occurs *via* an electrogenic pathway involving Mitochondrial Calcium Uniporter (MCU)^8^. The increase in intra-mitochondrial calcium concentration induces the opening of mitochondrial permeability transition pore (mPTP), which plays an important role in the induction of apoptosis.

To date, knowledge of the action of metformin on mitochondria has been limited to its effect on energy stress, which sensitizes cancer cells to apoptosis^6^. Here, we studied the effects of biguanides on mitochondria. We found that metformin and phenformin induced mitochondrial swelling and disorganization of the cristae of the inner mitochondrial membrane (IMM) *in vitro* and *in vivo*. Metformin induced ER stress and triggered the release of calcium from the ER, thus leading to mitochondrial calcium uptake. We also demonstrated that metformin inhibited the opening of mPTP. Finally, in response to metformin, mitochondrial biogenesis was induced in cancer cells. These findings suggest a novel role for metformin in triggering pro-survival responses in cancer cells.

## Results and Discussion

**Figure 1:**
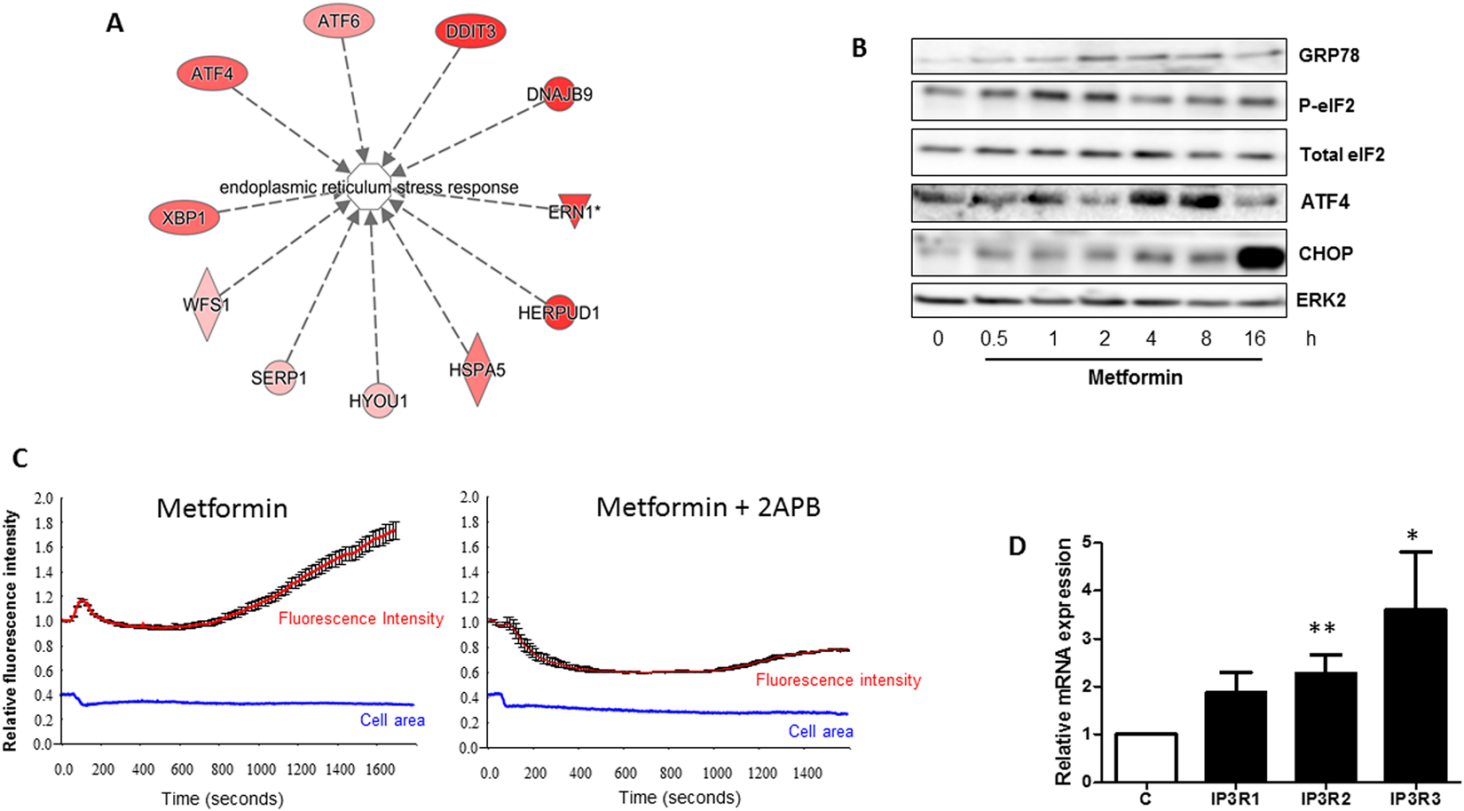
Metformin induces ER stress and calcium release from the ER. (**A**) List of genes related to the ER stress pathway and significantly upregulated in LNCaP cells treated with 5 mM metformin for 14 h. **(B)** Immunoblotting of LNCaP cells treated with 5 mM metformin for the indicated times by using antibodies directed against the indicated proteins implicated in ER stress response. The immunoblot is representative of three independent experiments. The blots represent (**C**) Relative fluorescence intensity of the Fluo-4AM probe, measured in LNCaP cells. 2-ABP (40 µM) was added 15 min before the addition of 5 mM metformin at time 0. The cellular area was monitored during the experiment (blue line). At least 30 single cells were monitored for each condition. The graph represents the mean fluorescence intensity +/− sem. (**D**) Quantification of IP3R mRNA in LNCaP cells treated with metformin for 24 h. p < 0.05 (*); p < 0.01 (**).

To better characterize the molecular targets of metformin in cancer cells, we performed microarray analysis in the prostate cancer cell line LNCaP, which was treated with metformin for 24 h. As expected, we observed an increase in the expression of genes encoding energy stress-related proteins such as the Regulated in Development and DNA Damage Responses 1 (REDD1) and Sestrin 2^9, 10^ (data not shown). Notably, metformin significantly increased the expression of genes implicated in the unfolded protein response (UPR) induced by ER stress, such as *XBP1*, *ATF4*, *ATF6* and *DNAJB9* (Fig. 1A). To further characterize and validate the ER stress response induced by metformin, we studied the expression of key players of the UPR by western blotting. After only 1 h, we observed an increase in the levels of Bip/GRP78 and P-eIF2 followed by an increase in the levels of ATF4 and CHOP/DDIT3, a downstream pro-apoptotic factor of the UPR, at 4 h (Fig. 1B and Figure 1-figure supplement 1).

**Figure 2:**
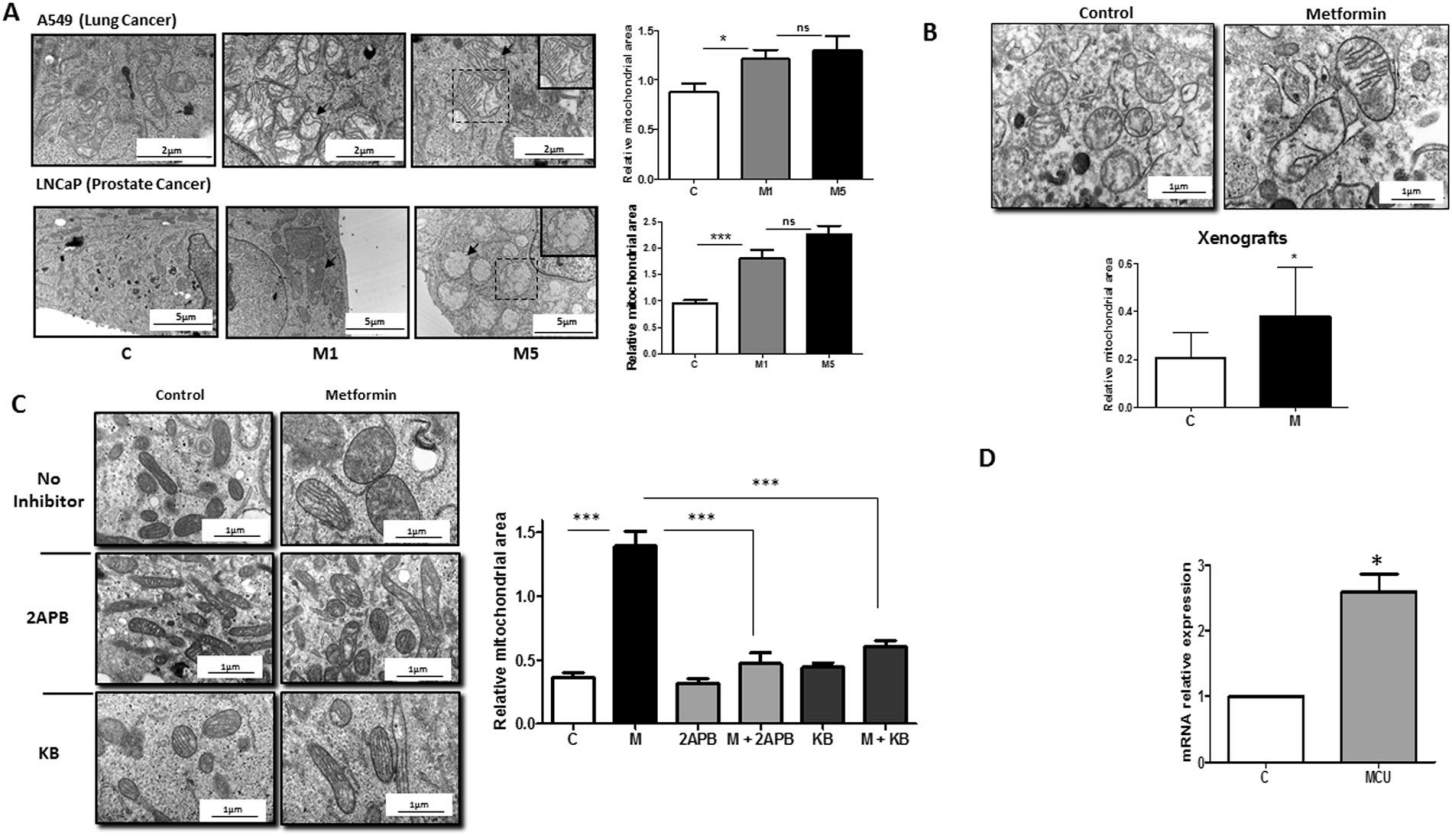
Metformin induces mitochondrial swelling in cancer cells *in vitro* and *in vivo*. (**A**) Electron microscopy (EM) of LNCaP (PCa) and A549 (lung cancer) cells treated with 1 and 5 mM metformin for 24 h. The graphs represent the mean of the relative mitochondrial area. An example of swollen mitochondria with modifications of the IMM is noted with an arrow. The inset shows a magnified view of a representative mitochondrion. (**B**) Electron microscopy images of representative mitochondria from tumor xenografts, and quantification of the relative mitochondrial area in the corresponding tumors. The error bars represent the standard error of the mean (sem). (**C**) Electron microscopy images of LNCaP cells treated with metformin and 40 µM 2-ABP or 10 µM KB-R7943 for 24 h. Quantification of the mitochondrial area is reported in the graph (left panel). For each condition, 60 to 100 mitochondria from several cells were analyzed by EM. (**D**) Quantification of MCU mRNA in LNCaP cells treated with metformin for 24 h. The differences are significant with p < 0.05 (*) and p < 0.001 (***).

The ER is the major calcium reservoir in cells. Thus, ER stress is often associated with modifications of the calcium flux within cells. Therefore, we analyzed the calcium flux in cells treated with metformin by using a Fluo-4AM probe. After metformin treatment, we observed a burst of fluorescence corresponding to the release of calcium into the cytoplasm (Fig. 1C, Movie S1) even in calcium-free medium that excluded the entry of extracellular calcium (data not shown). To confirm that metformin triggers the release of calcium from the ER, we treated the cells with 2-aminoethoxydiphenyl borate (2-APB), an inhibitor and antagonist of IP3R (a calcium releasing channel of the ER)^11^. Treatment with 2-APB hampered the changes in calcium flux induced by metformin, thus demonstrating that the metformin-induced calcium flux originates in the ER (Fig. 1C). Accordingly, we also observed a significant increase in the expression of mRNA encoding for IP3R2 and IP3R3 after metformin treatment (Fig. 1D). The mitochondria display essential calcium buffering properties^12^; therefore, we hypothesized that calcium release from the ER might be buffered by the mitochondria. To determine the effects of metformin on the mitochondria, we performed electron microscopy analysis in several cancer cells lines (LNCaP, DU145, A375 and A549). We treated the cells with 1 or 5 mM metformin, commonly used concentrations that inhibit proliferation by 20% to 60%^3, 13, 14^. Metformin and phenformin induced swelling of the mitochondria and a profound disorganization of the IMM and cristae without causing visible modifications of the outer mitochondrial membrane (OMM) (Fig. 2A and Figure 2-figure supplement 1). Twenty-four hours after metformin withdrawal, the size of the mitochondria had partially returned to normal, and the organization of the cristae was similar to that of the untreated cells (Figure 2-figure supplement 2). We also demonstrated that the effect on mitochondrial swelling occurred as early as 4 h after the addition of metformin (Figure 2-figure supplement 3). Of note, metformin did not alter the phenotype of the mitochondria in the normal epithelial prostate cell line P69 (Figure 2-figure supplement 4). Finally, we did not observe modifications of the mitochondrial ultrastructure in cancer cells treated with AICAR, another energy disruptor and activator of AMPK **(**data not shown).

We then asked whether these modifications occur *in vivo*. We studied the mitochondria in tumor xenografts of LNCaP cells implanted in SCID mice that were given 100 mg/kg of metformin in drinking water for 8 weeks. As shown previously^13^, metformin administration resulted in the expected antitumoral effects (Figure 2-figure supplement 5), and the mitochondria were significantly enlarged in the xenografts from the metformin-treated mice compared with the mitochondria in the control group (Fig. 2B). Similar results were obtained in TRAMP mice that were given 100 mg/kg of metformin for 6 months. In the treated mice, metformin efficiently reduced tumor growth and increased the mitochondrial area in the tumor cells (Figure 2-figure supplement 5,6).

Whether the release of calcium is a consequence of ER stress or the inducer remains an open question. Thapsigargin and tunicamycin can successfully induced ER stress and then the release of calcium, its accumulation in the mitochondria and finally mitochondrial swelling^15^. Previous studies have shown that metformin induces ER stress in the prostate cancer cell line C4-2B through the induction of miR-708-5p^16^, but a direct link between metformin and mitochondrial calcium homeostasis has never been demonstrated. A direct action of metformin on IP3R, which could trigger the release of calcium and consequently ER stress cannot be excluded, but there is no evidence in the literature of such a mechanism of action. The observation that metformin modulates intracellular calcium flux is novel, although a recent study based on transcriptomic and proteomic analyses in breast cancer cell lines demonstrated that metformin induced a significant upregulation of pathways involved in calcium signaling^17^.

To validate the direct relationship between the release of calcium from the ER and the swelling of mitochondria, we inhibited IP3R with 2-APB and treated LNCaP cells with metformin. While 2-APB alone did not affect the ultrastructure of mitochondria, it significantly blocked the swelling of mitochondria induced by metformin (Fig. 2C). To further confirm the implication of calcium, LNCaP cells were treated with two well-characterized chemical inhibitors of mitochondrial calcium uniporter (MCU), KB-R7943^18^ and RU-360^19^. Treatment with the inhibitors alone had no effect on the mitochondrial phenotype, whereas in the presence of metformin, KB-R7943 significantly inhibited mitochondrial swelling (Fig. 2C) and RU-360 preserved the organization of IMM and cristae (Figure 2-figure supplement 7). The transfer of calcium from the ER to the mitochondria involves IP3R (for the release from the ER), VDAC1 at the OMM and MCU at the IMM. We found that metformin increased the levels of IP3R1, IP3R2 IP3R3 and MCU mRNA as well as VDAC1 protein (Figs 1D, 2D and 4B). Altogether, these results demonstrate that there is a direct relationship between the release of calcium and mitochondrial swelling.

**Figure 3:**
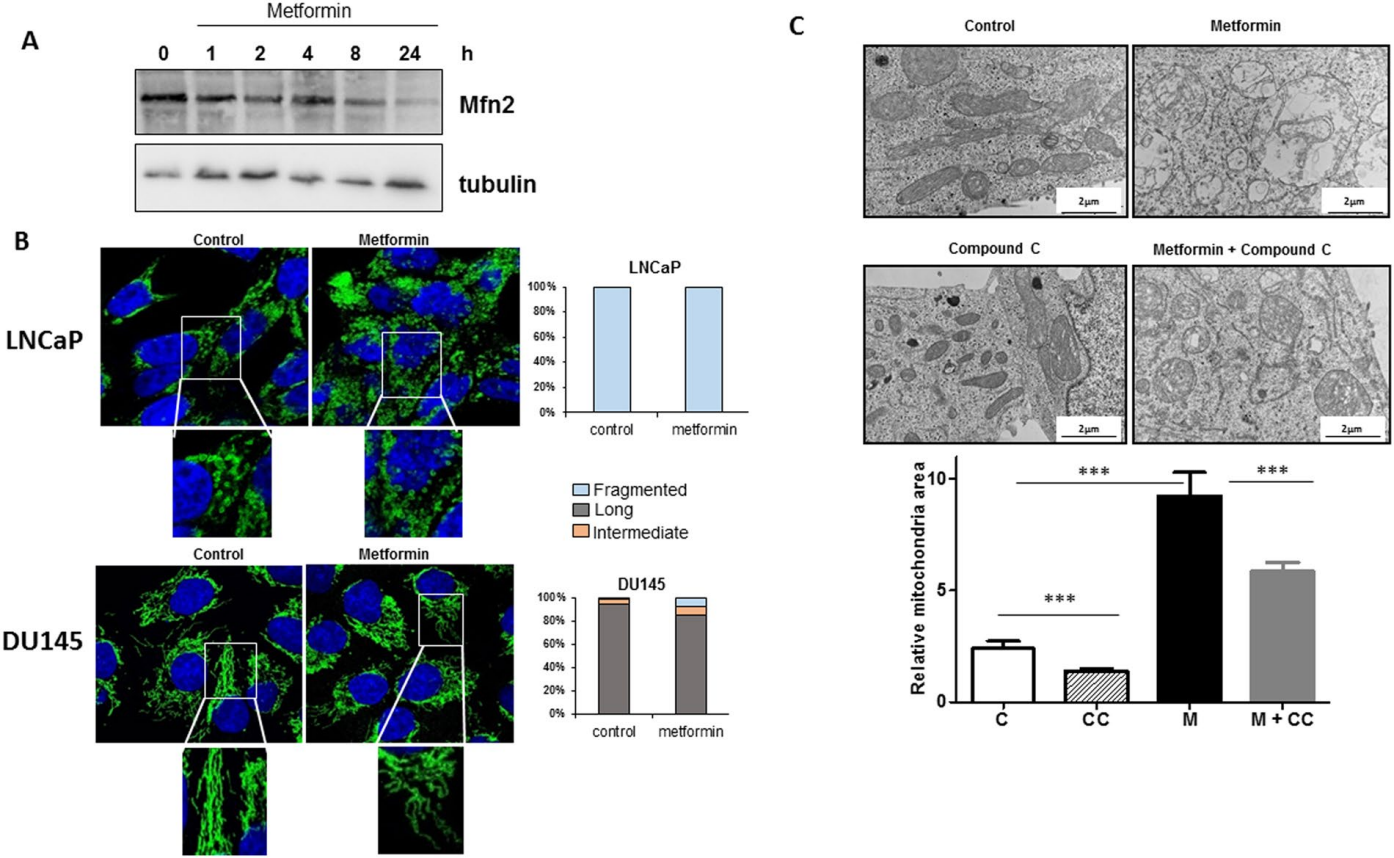
Metformin does not induce fission of the mitochondrial network, and mitochondrial swelling is partially dependent on AMPK. (**A**) LNCaP cells were treated with metformin for the indicated times, and Mfn2 expression was monitored at different time by immunoblotting. (**B**) LNCaP and DU145 cells were treated for 24 h with 5 mM metformin and stained with Tom20 for immunofluorescence analysis. Representative IF images are shown with the quantification of the morphology of the mitochondrial network. The data from the counting of at least 100 cells are shown. (**C**) LNCaP cells were treated with 5 mM metformin for 24 h in the presence or absence of 20 µM of compound C (CC). Representative electronic microscopy images are shown, and the graph represents the mean of the relative mitochondrial area. For each condition, 60 to 100 mitochondria from several cells were analyzed by EM, *p* < 0.001 (***).

The ability of mitochondria to swell in response to different stresses is a biological process that has been known for decades^20^. Hypoxia, through HIF-1α, induces the enlargement of mitochondria, resulting from an atypical fusion of mitochondria due to an increase in the expression of mitofusin (Mfn)^21^. Our previous unpublished data showed that metformin did not alter the expression of HIF-1α in LNCaP cells. In addition, we demonstrate here that metformin decreased the expression of Mfn2 (Fig. 3A), suggesting that the mechanism implicated in mitochondrial swelling upon metformin treatment and hypoxia are different. Interestingly, invalidation of Mfn2 in the cerebellum impaired mitochondrial fusion and lead to aberrant mitochondrial ultrastructure and enlargement of mitochondria in Purkinje cells^22^. Other well-known inducers of mitochondrial swelling are perturbator of intracellular K + homeostasis (valinomycin) and more importantly calcium^17^.

Energy disruptors and activators of AMPK such as rotenone and AICAR lead to the fragmentation of the mitochondrial network^23^. Because metformin is an energy disruptor and activator of AMPK, we studied the mitochondrial network by confocal imaging by using the mitochondrial marker Tom20. LNCaP cells have a disconnected mitochondrial network under basal conditions, and metformin did not induce the fragmentation of the mitochondrial network in DU145 cells (Fig. 3B). However, metformin decreased the expression of mitofusin 2 (Mfn2), a major regulator of mitochondrial fusion (the opposite process from mitochondrial fission^24^), thus suggesting that although we did not observe a clear fragmentation phenotype induced by metformin, mitochondrial fusion may have been altered by metformin. We and others have found that some of the biological effects of metformin are not mediated by AMPK^9, 25^. Therefore, we asked whether AMPK might be involved in the ultrastructural modifications induced by metformin. To this end, we treated cells with compound C (CC), a commonly used AMPK inhibitor. The inhibitor alone diminished the mitochondrial area and partially, but significantly, reversed the swelling and ultrastructural modifications induced by metformin (Fig. 3C). Altogether, our results demonstrated that metformin does not induce fragmentation of the mitochondrial network and exerts effects on mitochondrial swelling, at least in part, *via* AMPK.

The relationship between energy stress and modifications of the mitochondrial network has been elucidated by the discovery of AMPK function in regulating mitochondrial fragmentation^23^. In this study, a short-term treatment (of minutes) with energy disruptors such as AICAR and rotenone induced fragmentation of the mitochondrial network. Metformin is a weak inducer of energy stress, as compared with AICAR and rotenone, thus potentially explaining the absence of mitochondrial fragmentation in prostate cancer cells. In addition, our study was performed 4 and 24 h after the addition of metformin. AMPK phosphorylated MFF, a receptor for Drp1 (a major regulator of mitochondrial fission). Interestingly, metformin induced the phosphorylation of MFF in hepatocytes^23^. It would be interesting to determine whether MFF is involved in the swelling of mitochondria.

**Figure 4:**
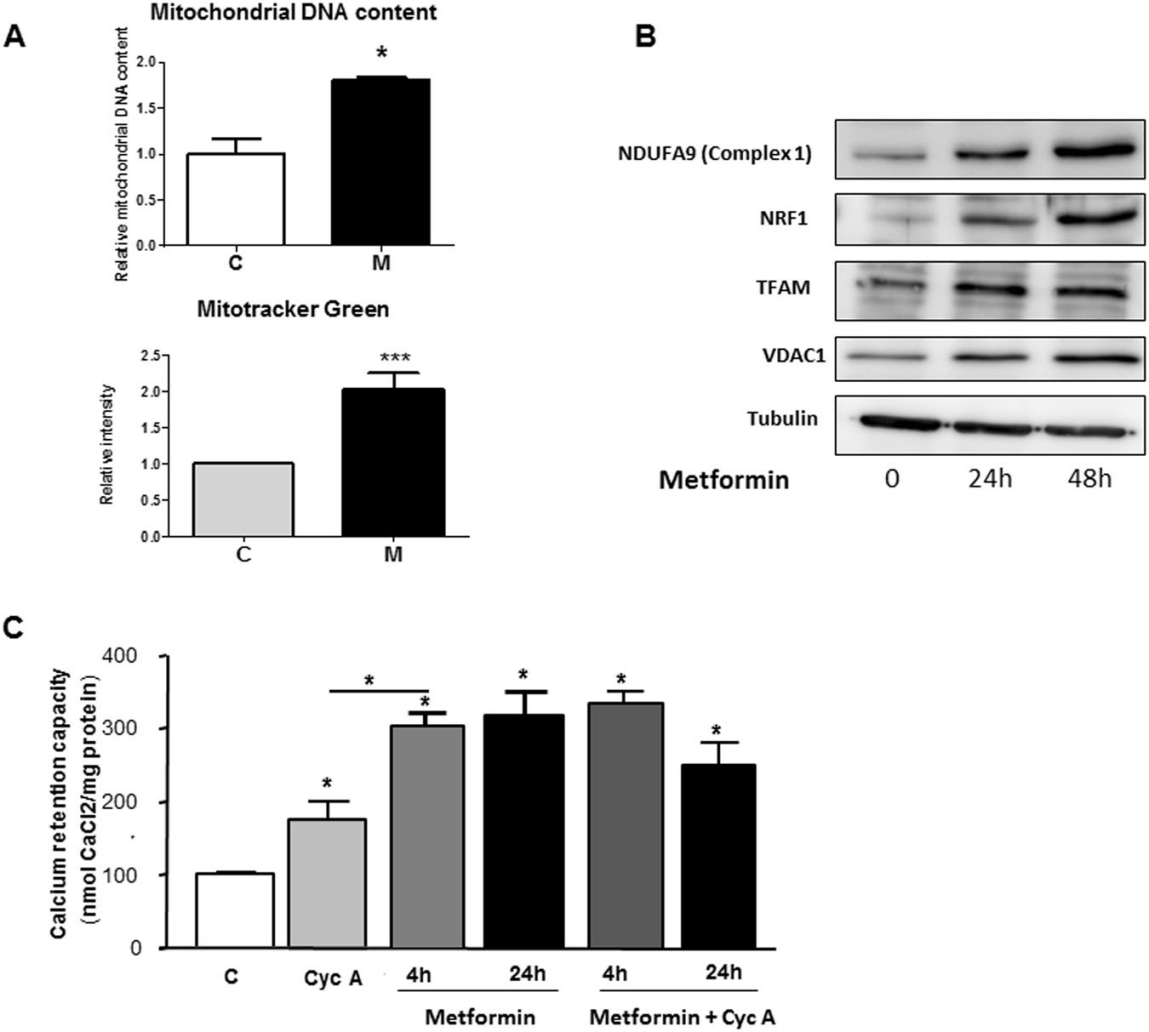
Metformin increases mitochondrial biogenesis in cancer cells and inhibits mitochondrial permeability transition pore. (**A**) Mitochondrial DNA content and quantification of MitoTracker Green in LNCaP cells treated with metformin for 24 h. The results were obtained from three independent experiments. *p* < 0.05 (*); *p* < 0.001 (***). (**B**) Immunoblotting analyses of proteins and transcription factors implicated in mitochondrial function, after treatment with 5 mM metformin for 24 and 48 h. (**C**) Calcium retention capacity measured after the incubation of LNCaP cells with 1 µM cyclosporin A for 4 h or 5 mM metformin for 4 h and 24 h.

To further characterize the effects of metformin on mitochondria, we monitored mitochondrial biogenesis. Within 24 h, metformin increased the mitochondrial mass, as shown by the augmentation of mitochondrial DNA/nuclear DNA ratio and increased signal of MitoTracker Green (Fig. 4A). A direct consequence was the increase in the mitochondrial protein NDUFA9 (Complex 1). This effect is driven by an increase in NRF1 and TFAM, two transcription factors that regulate mitochondrial biogenesis (Fig. 4B). Altogether, these results demonstrated that metformin promotes mitochondrial biogenesis in prostate cancer cells. Because mitochondria are essential for supplying energy and play a central role in the metabolism of glutamine or synthesis of purines and pyrimidines in cancer cells, it would be interesting to investigate the importance of mitochondrial biogenesis in the metabolism, survival, and chemotherapy resistance of cancer cells.

The intra-mitochondrial concentration of calcium determines the opening of mPTP, and metformin has been shown to inhibit mPTP in INS-1 (isolated insulinoma cells), HeLa and KB cells and in cardiomyocytes^26–28^. To determine the effect of metformin on mPTP in cancer cells, we studied the Calcium Retention Capacity (CRC) of mitochondria in LNCaP and A375 cells treated with metformin. We found that metformin significantly increased CRC of mitochondria after 4 h and 24 h in both cell lines (Fig. 4C and Figure 4-figure supplement 1,2). Interestingly, the effect of metformin was stronger than that of cyclosporin A, a specific inhibitor of mPTP, and co-treatment with cyclosporin A and metformin had similar effects to those of metformin alone in LNCaP cells (Fig. 4C). Of note, we observed a significant decrease in the mRNA levels of cyclophilin D (Figure 4-figure supplement 3), a putative component of mPTP whose downregulation has been implicated in the retention of calcium in mitochondria^29^. Thus, the inhibition of the opening of mPTP by metformin may explain the weak pro-apoptotic potential of metformin.

During apoptosis, the opening of mPTP leads to the release of cytochrome c and allows the entry of ions and water into the matrix resulting in mitochondrial swelling^30^. The closure of mPTP with cyclosporine A, an inhibitor of cyclophilin D, reverses this swelling. In contrast with those results, we found that metformin inhibits the opening of mPTP and leads to mitochondrial swelling. We suggest that this discrepancy may be due to the massive influx of calcium into the mitochondria, probably through VDAC1 and MCU, which causes influx of water and osmotic swelling independently of mPTP. This hypothesis was supported by the electron microscopy data showing a clearer density of the matrix (Figs 2A and 3C and Figure 2-supplement 1 and 2) and by the increase in VDAC1 and MCU expression observed in the metformin-treated cells.

**Figure 5:**
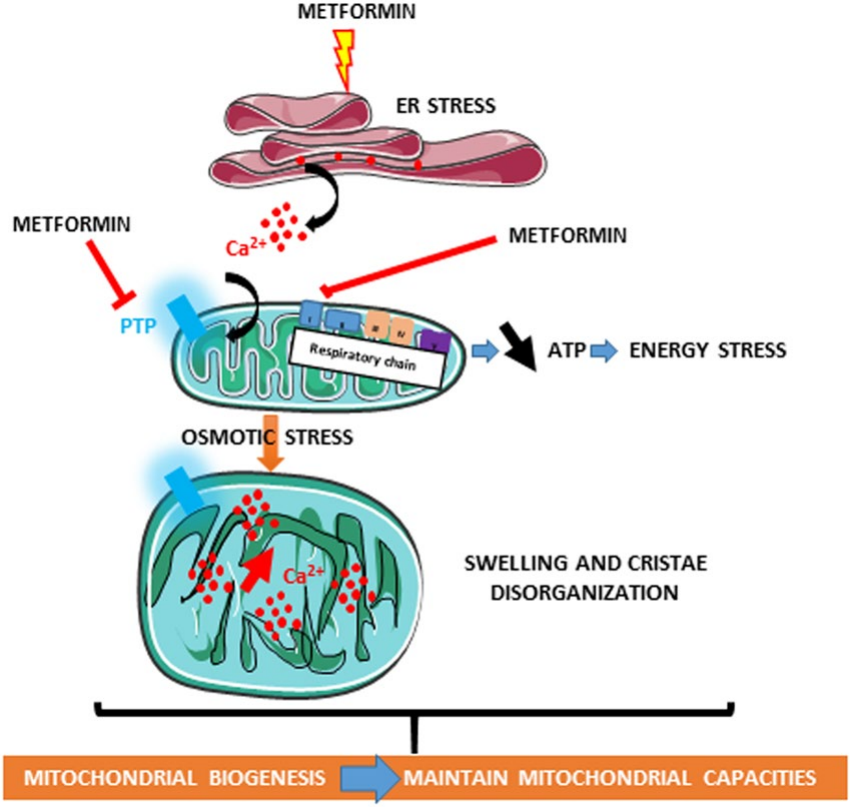
Schematic representation of the effects of metformin on mitochondrial biology in cancer cells. The illustrations were created by adopting templates from Servier Medical Arts (http://www.servier.fr/smart/banque-dimages-powerpoint) which are free for use under the terms of the Creative Commons 3.0 License.

One of the novel findings in our study is that metformin modifies mitochondrial homeostasis in a different manner from acting directly on the respiratory chain complexes. The accumulation of calcium within the mitochondria regulates the intrinsic function of the organelle, including the production of ATP^31^. Three enzymes of the matrix are regulated by calcium: pyruvate dehydrogenase, α-ketoglutarate and isocitrate-dehydrogenase^32^. Activation of these enzymes increases the NADH/NAD ratio and hence the flow of electrons through the respiratory chain, thus increasing ATP production. Metformin inhibits complex 1 and decreases ATP production. Therefore, we postulate that similarly to the compensatory increase of glycolysis observed in response to metformin^6^, the increase in calcium influx may preserve the energetic status of cancer cells. Likewise, the induction of mitochondrial biogenesis may preserve the mitochondrial capacity, thereby increasing the calcium buffering abilities of the cells (Fig. 5). Together with the inhibition of mPTP opening, these biological responses protect cancer cells against apoptosis. The concept of the protective effect of metformin in normal cells is not new, but this effect is not extensively documented in cancer cells. Metformin has been shown to protect primary cortical neurons and cardiomyocytes from apoptosis^33, 34^. It also protects the Isolated insulinoma cell line INS-1β from apoptosis induced by high concentrations of glucose, a condition that induces mPTP opening and apoptosis^27^. This protective effect may seem controversial, given the antitumoral effect of the drug. However, we and others have shown that metformin is a stronger inhibitor of cell cycle than it is an inducer of apoptosis^13, 35, 36^.

The relationship between complex 1 and mPTP is complicated. Previous studies have suggested that the inhibition of complex 1 is related to the effect of metformin on mPTP^27, 28^. It has been proposed that complex 1 interacts with mPTP and modulates its opening through a mechanism similar to the displacement of cyclophilin D by cyclosporin A^27^. However, Batandier *et al*. have shown that the opening of mPTP directly affects electron transfer through complex 1^37^.

In conclusion, we identify a new mechanism of action for metformin involving interference with mitochondrial biology through modification of calcium flux. The inhibition of mPTP opening and increase in mitochondrial biogenesis are consistent with the anti-apoptotic and pro-survival response that should be considered in the future for the potential use of metformin in cancer therapy.

## Methods

### Cell lines and culture conditions

The cell lines were purchased from ATCC and authenticated by ATCC (Manassas, VA, USA). LNCaP cells were cultured in RPMI 1640 medium; DU145, A375 and A549 cells were cultured in DMEM (Invitrogen, Carlsbad, CA, USA) containing 4.5 g/L glucose supplemented with 10% fetal bovine serum (FBS) and 100 units/mL penicillin at 37 °C and 5% CO_2_. The cell line P69 was derived by immortalization of human primary prostate epithelial cells with simian virus-40 T antigen^38^ and grown in RPMI 1640 with 10% FBS.

### Chemicals

Metformin and phenformin were suspended in culture medium (without FBS and penicillin) and used at the respective concentrations of 5 mM and 100 µM, unless stated otherwise. Metformin, phenformin, 2-APB, KB-R7943 and cyclosporin A were purchased from Sigma-Aldrich. RU-360 was purchased from Merck Millipore.

### Animal experiments

TRAMP mice were purchased from the Jackson Laboratory (stock # 003135). Metformin (100 g/kg/day) was dissolved in drinking water and administered to 6-week-old mice until the day of sacrifice at 8 months. For generating the tumor xenografts, LNCaP cells were inoculated subcutaneously (s.c.) with BD Matrigel™ matrix (BD Biosciences, Bedford, MA, USA) in five-week-old male SCID mice in both flanks (10^6^ cells per site). Treatment with metformin in drinking water (100 g/kg/day) was started 3 days after tumor inoculation in mice. Tumor size was measured twice weekly for 8 weeks with a caliper, and the tumor volume was calculated with the formula π/ 6 × *A* × *B* ^2^ (*A* = larger diameter of the tumor, *B* = smaller diameter of the tumor) as previously described^13^. The animal experiments were carried out in accordance with French law and were approved by the local institutional ethics committee CIEPAL (Comité Institutionnel d’Éthique Pour l’Animal de Laboratoire-Azur).

### Electron microscopy

The cells were fixed with 1.6% glutaraldehyde in 0.1 M phosphate buffer at room temperature (RT) and then for 16 h at 4 °C. The samples were rinsed with the same buffer, then post-fixed with 1% osmium tetroxide and 1% potassium ferrocyanide in 0.1 M cacodylate buffer for 1 h at RT. The cells were rinsed with distilled water and embedded in epoxy resin and conventionally processed for thin-sectioning and observed with a JEM1400 transmission electron microscope (Jeol, Japan) equipped with a Morada CCD camera (Olympus SIS, Germany). The mitochondrial area was calculated by two different investigators in a blinded manner. The measurement was performed as follows: for each condition, we delineated the area occupied by individual mitochondrion in at least 5 cells (2–3 sections per cells) with ImageJ. This represented 60 to 100 mitochondria per condition; the means +/− sem are reported in the graphs.

### Calcium Retention Capacity (CRC)

Extra-mitochondrial calcium measurement was performed with a spectrofluorometer (excitation 500 nm; emission 530 nm) as previously described^39^. Briefly, cells (equivalent to 800 µg of proteins) were added to 2 ml of CRC medium (150 mM sucrose, 50 mM KCl, 2 mM KH_2_PO_4_, 20 mM Tris/HCl and 5 mM Tris succinate). This medium was supplemented with 40 µM digitonin (to permeabilize the cells) and Calcium Green-5N (detection probe). The CRC measurements were performed in basal conditions or in the presence of the pharmacological compounds 1 µM cyclosporin A or 5 mM metformin. After 2 min of incubation, CRC was measured by addition of CaCl_2_ pulses (40 nmol or 20 nmol calcium) every 2 min until the opening of mPTP.

### Calcium flux measurements

Real-time calcium measurements were performed in LNCaP cells incubated in a physiological Tyrode solution (140 mM NaCl, 4 mM KCl, 1 mM MgCl_2_, 5 mM HEPES, 1.8 mM CaCl_2_ and 11 mM glucose, pH 7.4). The cells were loaded with the Fluo-4AM dye at 37 °C for 15 min and observed with a LSM510 Meta microscope (Zeiss). The experiment was independently performed twice, and 32 cells were monitored for each condition and each experiment.

### Microarray experiments

Pan-genomic microarrays were printed using the human RNG/MRC oligonucleotide collection, as previously described^40^. The experimental data and associated microarray designs have been deposited in the NBCI Gene Expression Omnibus (GEO) under series GSE83673 and platform record GPL 1456. Normalizations were performed using the Limma package available from Bioconductor (http://www.bioconductor.org). Intra-slide and inter-slide normalizations were performed using the Print Tip Loess and quantile methods, respectively. The means of ratios from all comparisons were calculated, and B-test analysis was performed using the Limma package available from Bioconductor. Differentially expressed genes were selected using a Benjamini-Hochberg correction of the p-value for multiple tests on the basis of a p-value below 0.05 and a fold-change cutoff (log Ratio > 0.7).

### Real-time quantitative PCR

The relative amount of mRNA was determined by quantitative RT-PCR using a SYBR Green Master Mix (Applied Biosystems)^9^. The primer sequences are available upon request.

### Western blot analysis

Cell extracts were prepared using lysis buffer^1313^. Immunoblotting was performed with antibodies against SDHA, NDUFA9 (Molecular Probes), NRF1, TFAM, VDAC1 (Santa Cruz Biotechnology) and α−tubulin (Sigma-Aldrich). Antibodies against ATF4, GRP78, total and phospho-eIF2α (Ser51) and CHOP/DDIT3 were purchased from Cell Signaling Technology. After incubation with the secondary antibodies, the membranes were washed 3 times, incubated with chemiluminescent HRP substrate Immobilon Western (Millipore) and scanned with a PXi gel imaging system.

### Statistical analysis

The significance of differences between the means of 2 groups was evaluated using Student’s t-tests.

## Electronic supplementary material

Supplementary data

Movie 1
