# Supplementary material for: The energy disruptor metformin targets mitochondrial integrity via modification of calcium flux in cancer cells
Source: Sci Rep. 2017 Jul 11;7:5040. doi: 10.1038/s41598-017-05052-2 (PMC5506014; doi:10.1038/s41598-017-05052-2)

SUPPLEMENTARY FIGURES

The energy disruptor metformin targets mitochondrial integrity via modifications of calcium flux in cancer cells

Camille LOUBIERE[1,2#], Stephan CLAVEL[1,2#], Jérôme GILLERON[1,2], Rania HARISSEH[3], Jéremy FAUCONNIER[4], Issam BEN-SAHRA[5], Lisa KAMINSKI[1,2], Kathiane LAURENT[1,2], Stéphanie HERKENNE [6,7], Sandra LACAS-GERVAIS[8], Damien AMBROSETTI[9], Damien ALCOR[1,2], Stéphane ROCCHI[2,10], Mireille CORMONT[1,2], Jean-François MICHIELS[9], Bernard MARI[11], Nathalie M. MAZURE[12], Luca SCORRANO[6,7], Alain LACAMPAGNE[4], Abdallah GHARIB[3], Jean-François TANTI[1,2], Frédéric BOST[1,2*]

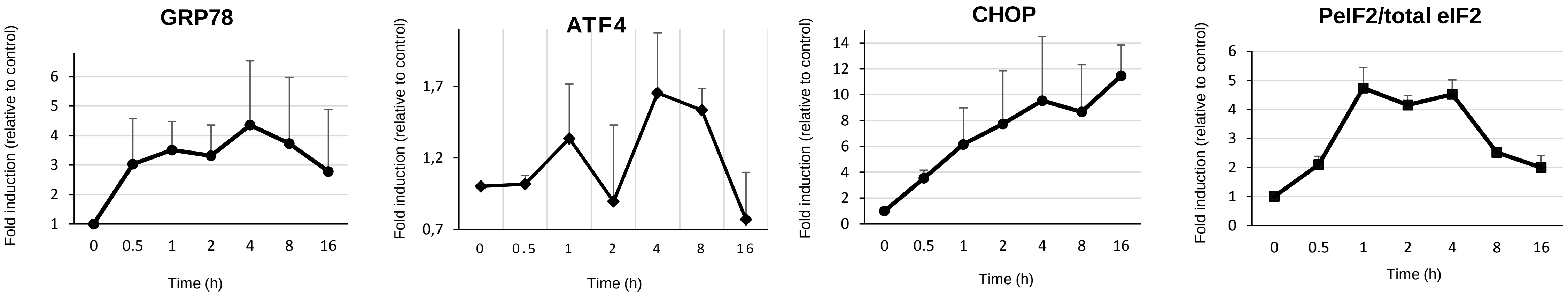

Figure 1- figure supplement 1: Quantification of the western blot presented in Figure 1. results are mean of three independent experiments.

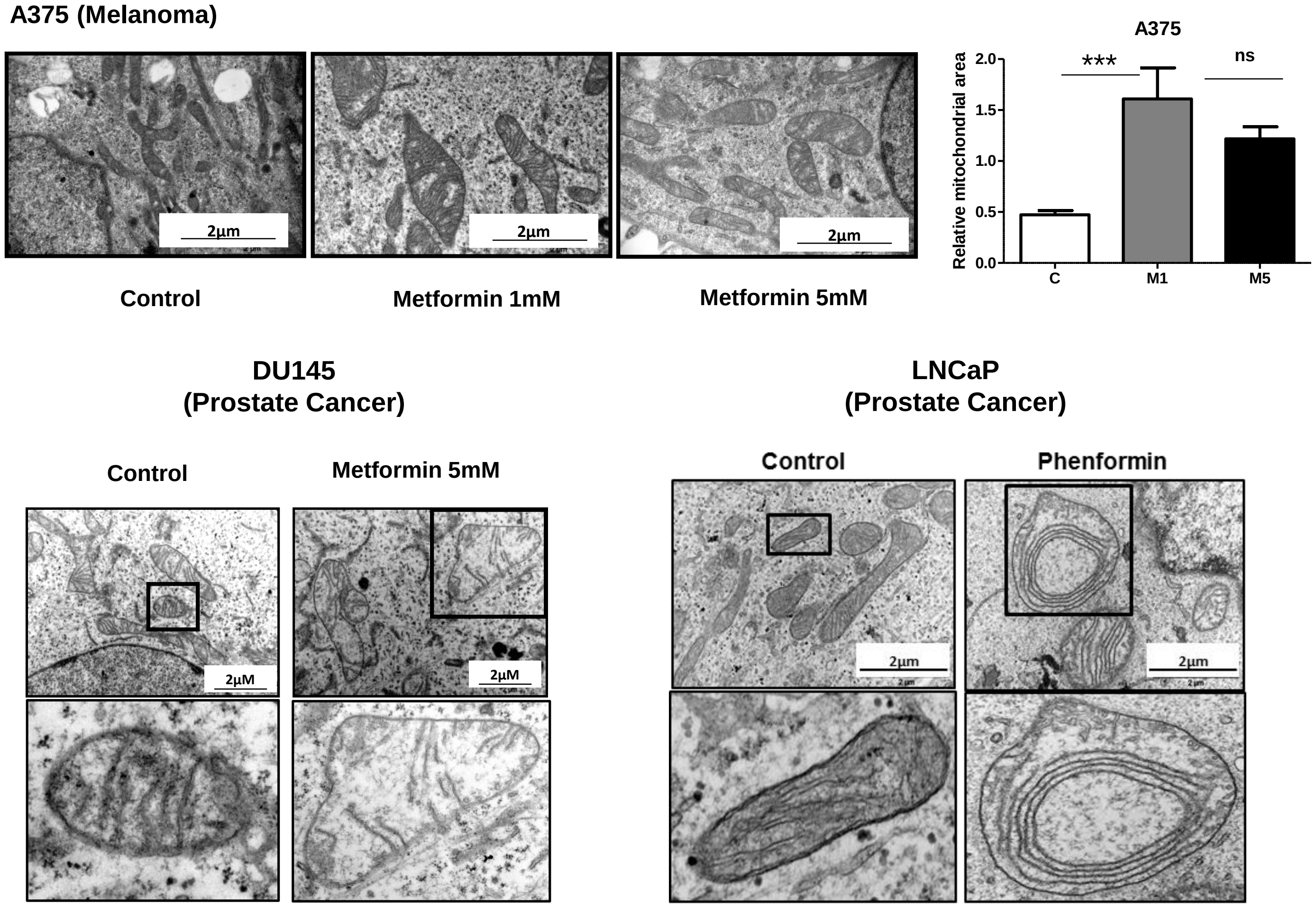

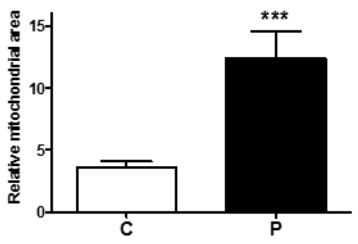

Figure 2- figure supplement 1: Metformin and Phenformin induces mitochondrial swelling and modifications of the phenotype of mitochondria in several cancer cell lines. Cancer cells are treated with 1 or 5 mM metformin or Phenformin for 24h and analyzed by electron microscopy.

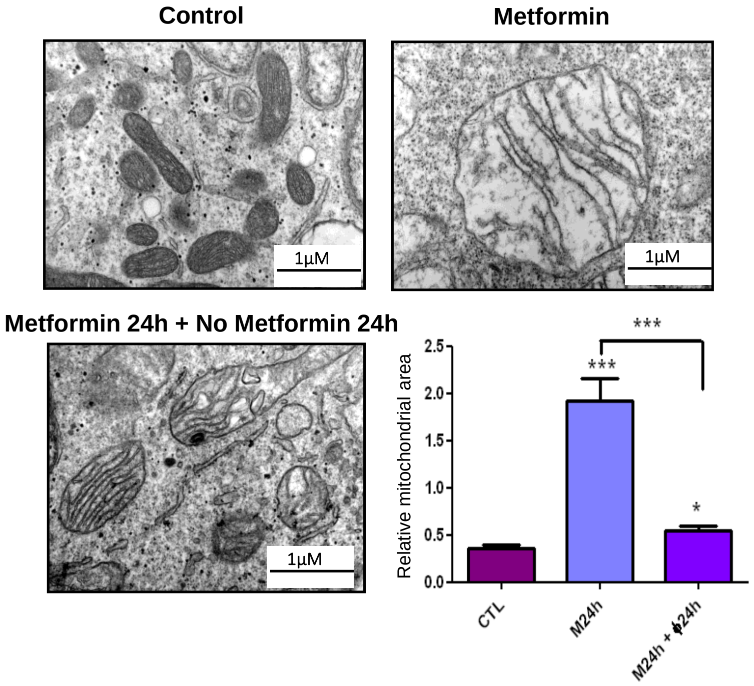

Figure 2- figure supplement 2 : The size of mitochondria partially returned to normal after 24h. LNCaP are treated for 24h with 5mM metformin and the media was replaced with fresh new media without metformin. The relative mitochondrial area is represenetd on the graph***p<0.01; *p<0.05

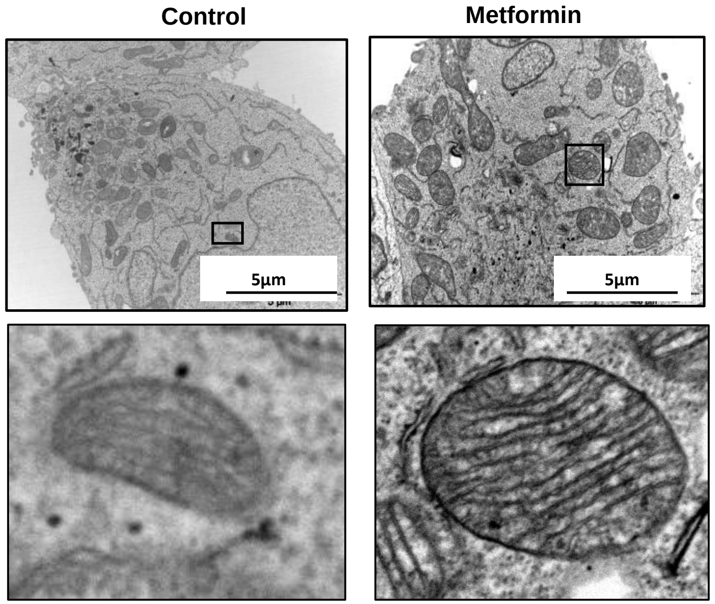

Figure 2- figure supplement 3: Metformin triggers the swelling of mitochondria after 4h of treatment with metformin. LNCaP cells are treated with 5 mM metformin for 4h before electron microscopy analysis.

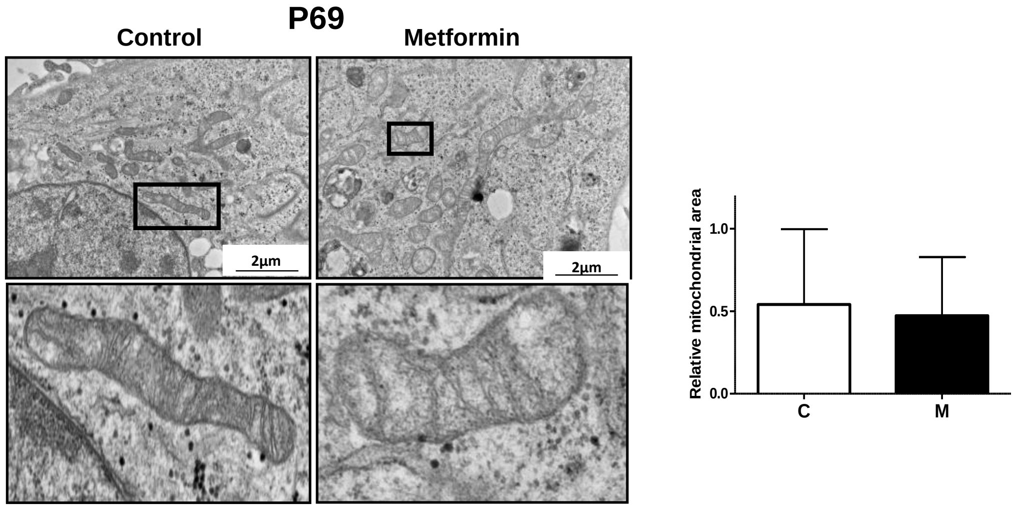

Figure 2- figure supplement 4 : Metformin does not alter mitochondrial ultrastructure in P69 cells. Cells are treated with 1 or 5 mM metformin for 24h and analyzed by electron microscopy.

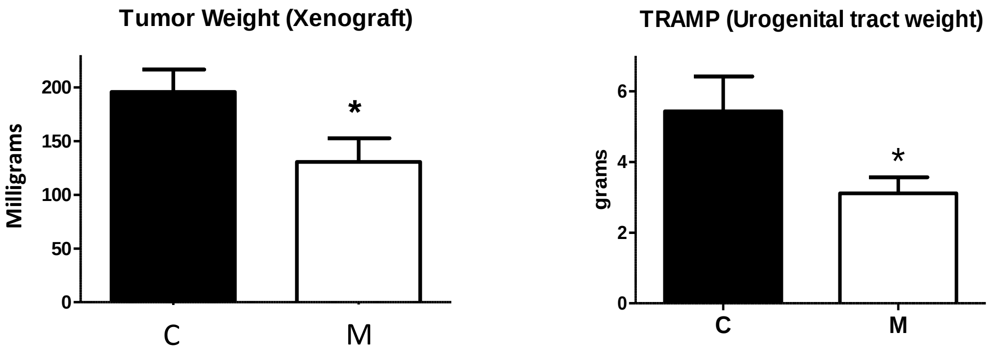

Figure 2- figure supplement 5. : Metformin inhibits tumor growth in a xenograft model using LNCaP cells and in TRAMP mice. Scid mice (n= 10 per group) are injected with LNCaP and receive 100mg/kg of metformin in drinking water. TRAMP mice (n=11 per group) were treated with the same dose of metformin. Tumor weight and Urogenital tract is monitored after 8 weeks and 6 month of treatment respectively.

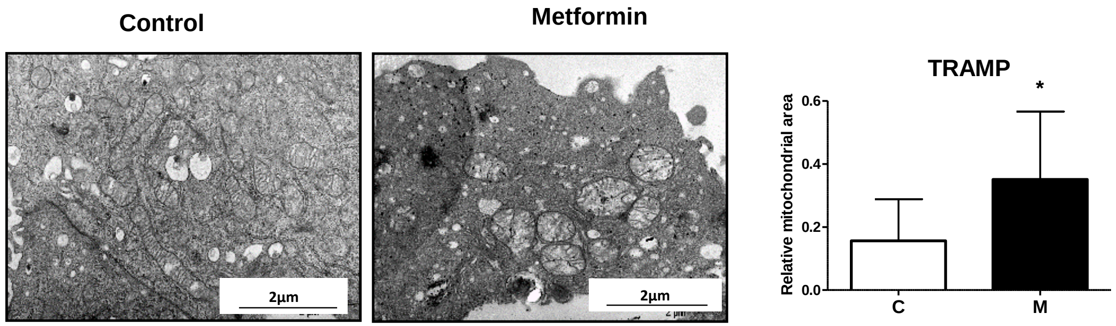

Figure 2- figure supplement 6: Metformin induces mitochondrial swelling in TRAMP tumors. Electron microscopy pictures of representative mitochondria from tumors (xenografts and TRAMP) and quantification of the relative area of mitochondria in the corresponding tumors.

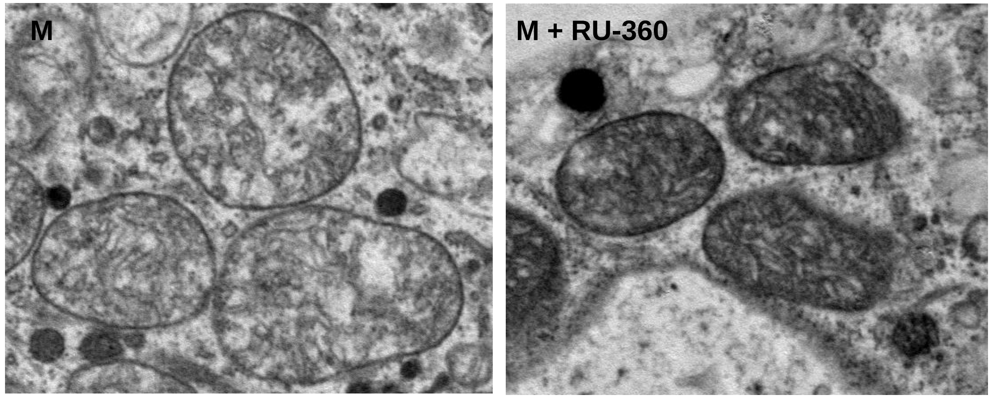

Figure 2- figure supplement 7: RU-360 reverses the disorganization of cristae induced by metformin. Representative pictures of mitochondria using electron microscopy. LNCaP cells are treated with 5 mM metformin alone or in presence of 10 µM RU-360 for 24h.

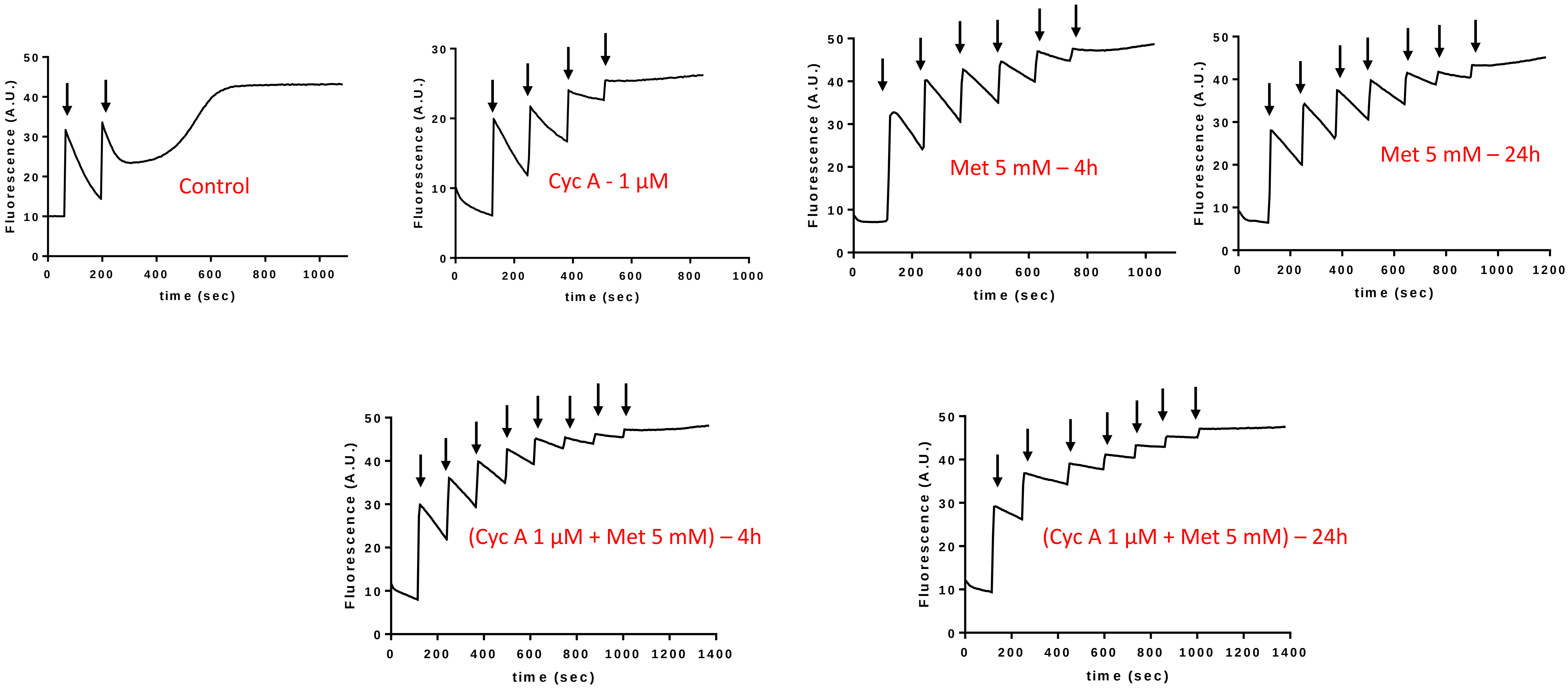

Figure 4 supplement 1: Original traces of flurorescence from a CRC experiement. The arrow indicated a pulse of calcium

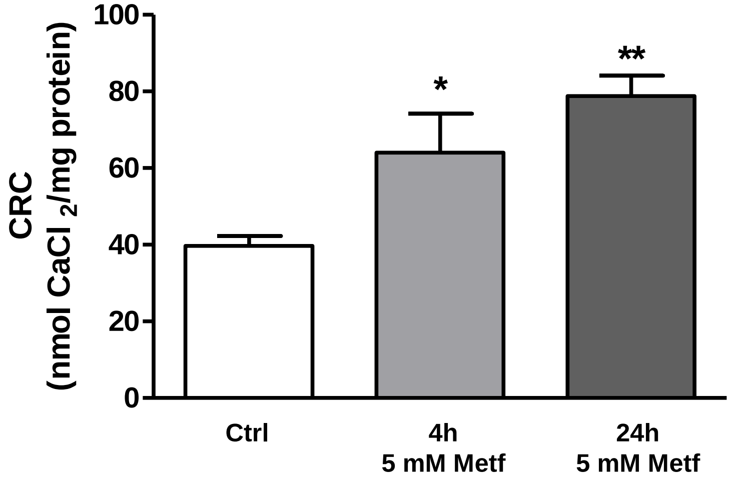

Figure 4- figure supplement 2 : Metformin increases CRC in A375 cells. Calcium retention capacity measured after the incubation of A375 cells with 5 mM metformin for 4h or 24h. * $p<0.05$; ** $p<0.01$

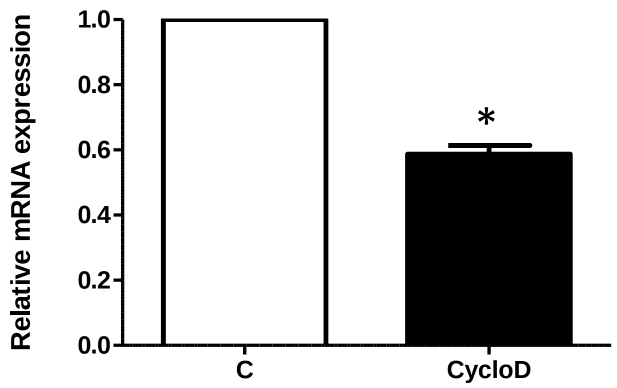

Figure 4- figure supplement 3 : Metformin significantly decreases the expression of Cyclophilyn D mRNA. LNCaP cells were treated with 5mM metformin for 24h

BLOTS Figure 1B

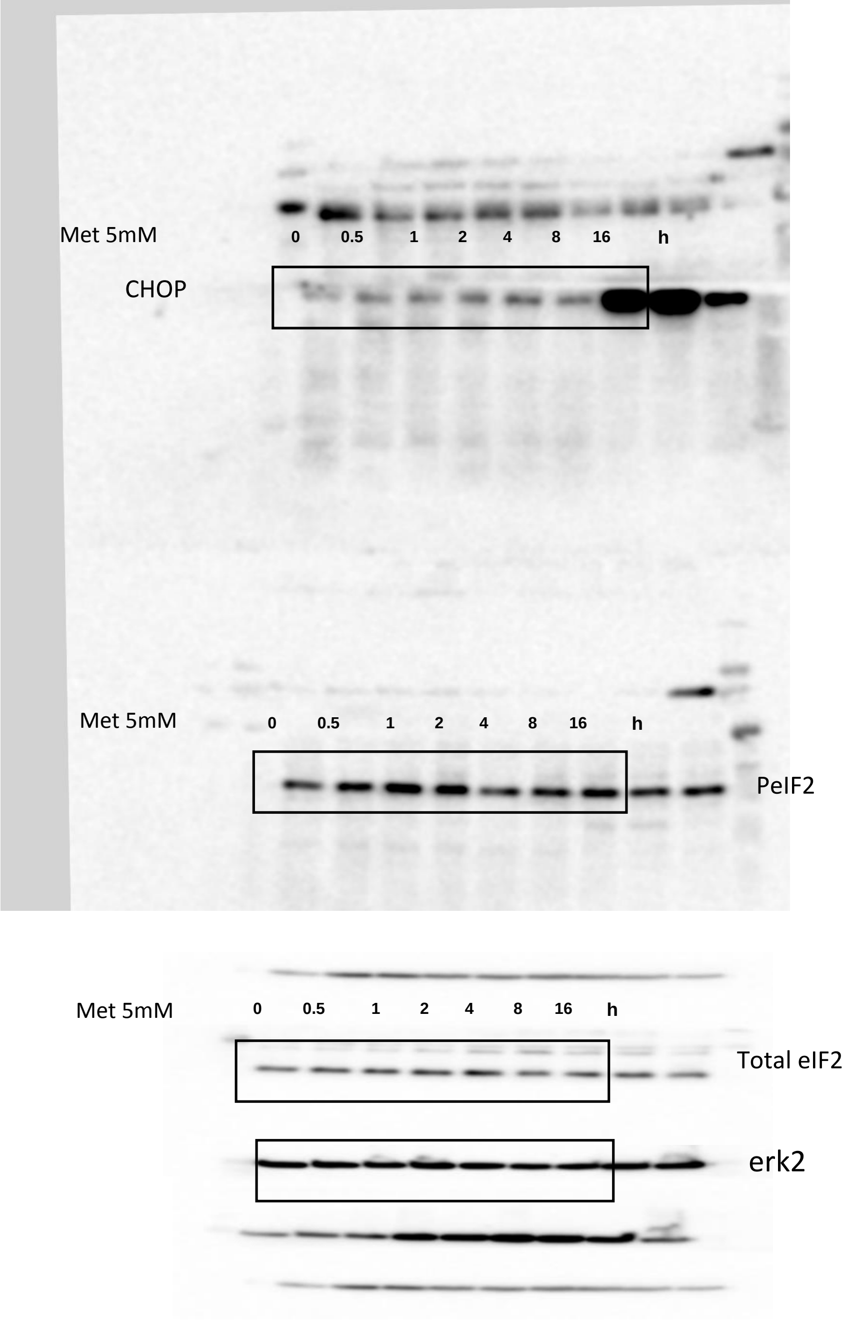

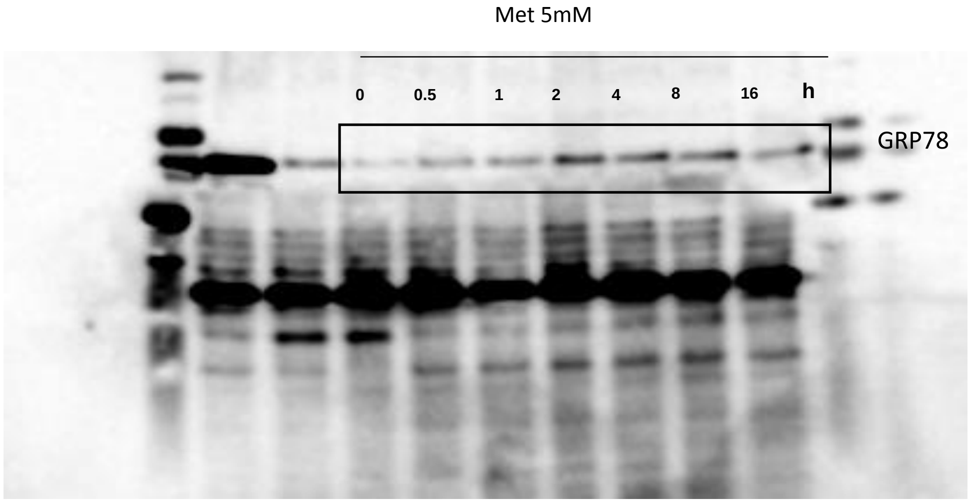

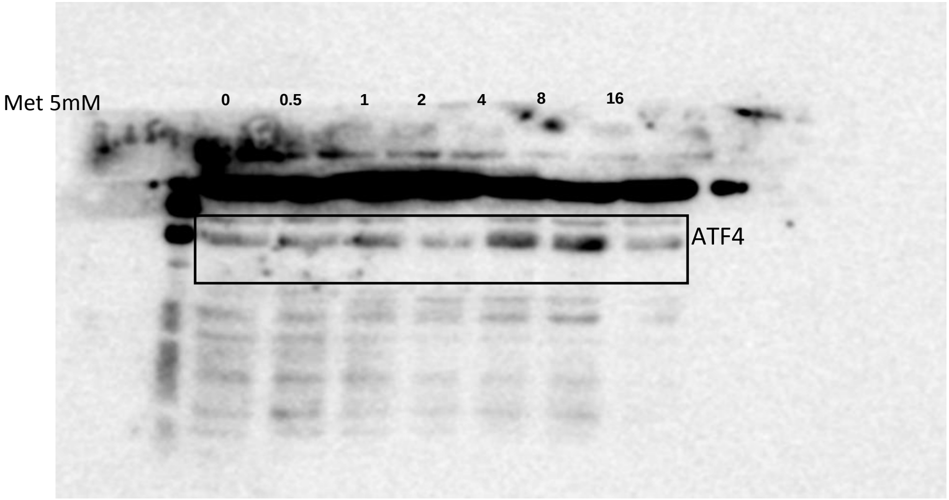

BLOTS Figure 4B

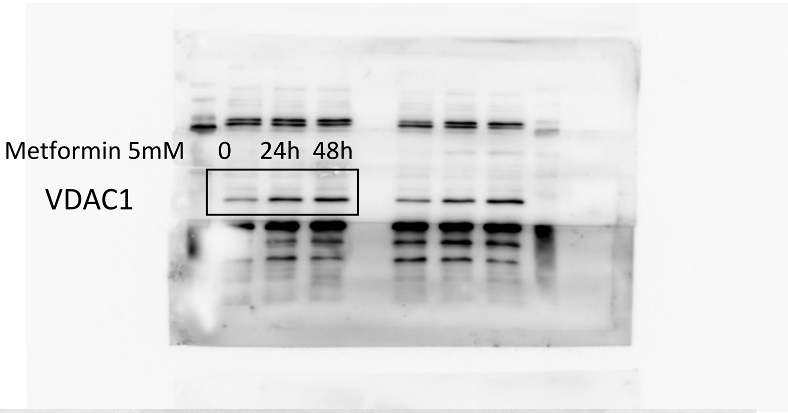

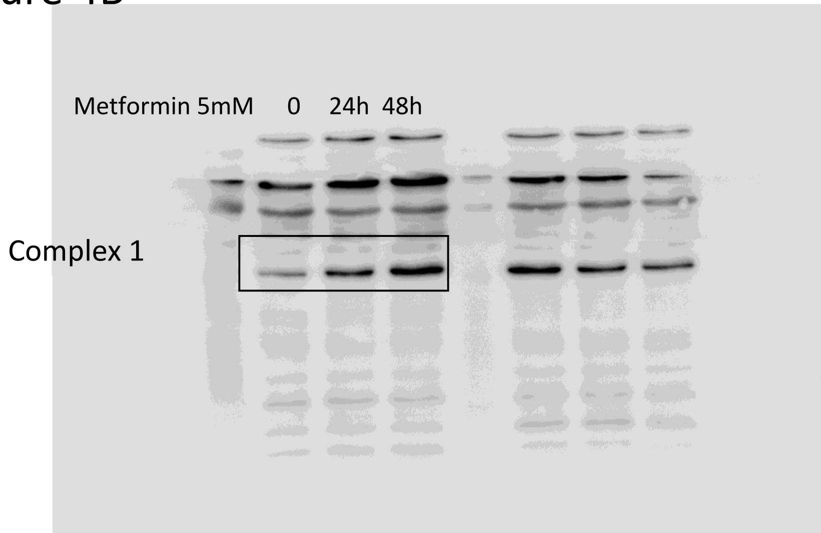

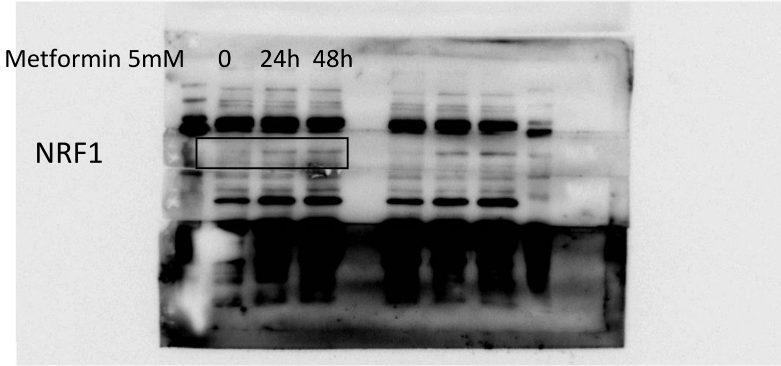

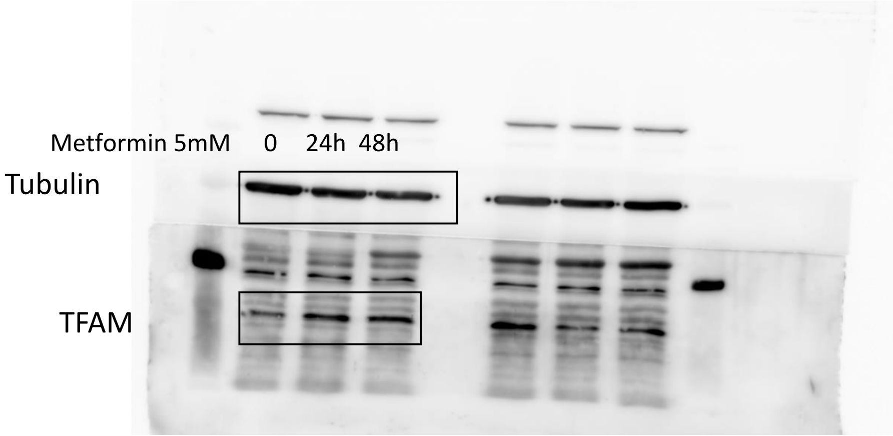

Supplement: Supplementary file 1 — Supplementary data [file 41598_2017_5052_MOESM1_ESM.pdf]
